# The role of ubiquitination and deubiquitination in cancer lipid metabolism

**DOI:** 10.3389/fonc.2025.1464914

**Published:** 2025-01-29

**Authors:** Yongkang Xu, Jiayu Zeng, Shumin Fu, Kan Liu, Ye Mao, Si Tao, Jianbing Wu

**Affiliations:** ^1^ Department of Oncology, the Second Affiliated Hospital of Nanchang University, Nanchang, Jiangxi, China; ^2^ Department of Pediatric Surgery, Jiangxi Provincial Children’s Medical Center, Jiangxi Maternal and Child Health Hospital, Nanchang, Jiangxi, China

**Keywords:** ubiquitination, deubiquitination, cancer, lipid, metabolism

## Abstract

The ubiquitin-proteasome system (UPS) is one of the main degradation systems within cells, catalyzing the tagging of proteins for degradation by ubiquitin molecules, which are then recognized and degraded by the proteasome. Lipid metabolism plays a crucial role in cellular energy metabolism and is closely associated with the occurrence and development of cancers. Recent research indicates that cancer lipid metabolism is regulated by intracellular proteins, including ubiquitination modifications. This review will explore the role of ubiquitination in regulating cancer lipid metabolism, summarize the latest research progress, and propose potential therapeutic strategies.

## 1 Introduction

Protein ubiquitination is a common and multifunctional post-translational protein modification, renowned for its ability to guide protein degradation via the ubiquitin-proteasome system (UPS) (1, 2). Ubiquitination and deubiquitination modify substrate proteins, regulating their lifespan and functionality, and play extensive roles in various physiological processes such as cell proliferation, apoptosis, autophagy, endocytosis, DNA damage repair, and immune response (3). Ubiquitination and deubiquitination are closely linked to cancer development, becoming a new target in cancer drug development (2). For example, LCL161, an IAP inhibitor, induces TNF-dependent apoptosis in multiple myeloma cells and enhances the anti-tumor immune response (4). The small molecule inhibitor SIM0501, which targets USP1, has FDA clinical approval and is planned for trials in advanced solid tumors. Thus, the study of protein ubiquitination is of great significance and may provide new opportunities for the diagnosis and treatment of cancer.

In the 1920s, Otto Warburg discovered that cancer cells preferentially use glycolysis for energy, even in the presence of oxygen, known as the “Warburg effect,” initiating cancer metabolism research (5). Tumor metabolic reprogramming primarily involves the upregulation of glycolysis, glutaminolysis, lipid metabolism, mitochondrial biogenesis, the pentose phosphate pathway, and other biosynthetic and bioenergetic pathways (6). Lipids metabolism are essential for constructing cell membranes, energy storage, and signaling in cellular processes, making the regulation of lipid metabolism crucial for maintaining cellular equilibrium (7). Dysregulation of lipid metabolism is one of the key metabolic changes in cancer. Recently, there has been increasing interest in the roles of ubiquitination and deubiquitination in regulating cancer cell metabolic reprogramming. This review will explore the effects of protein ubiquitination on cancer and its association with alterations in lipid metabolism. Our focus will be on existing research findings and prospective research avenues to enhance opportunities for cancer diagnosis and treatment advancements.

## 2 Ubiquitination and deubiquitination

**Figure 1:**
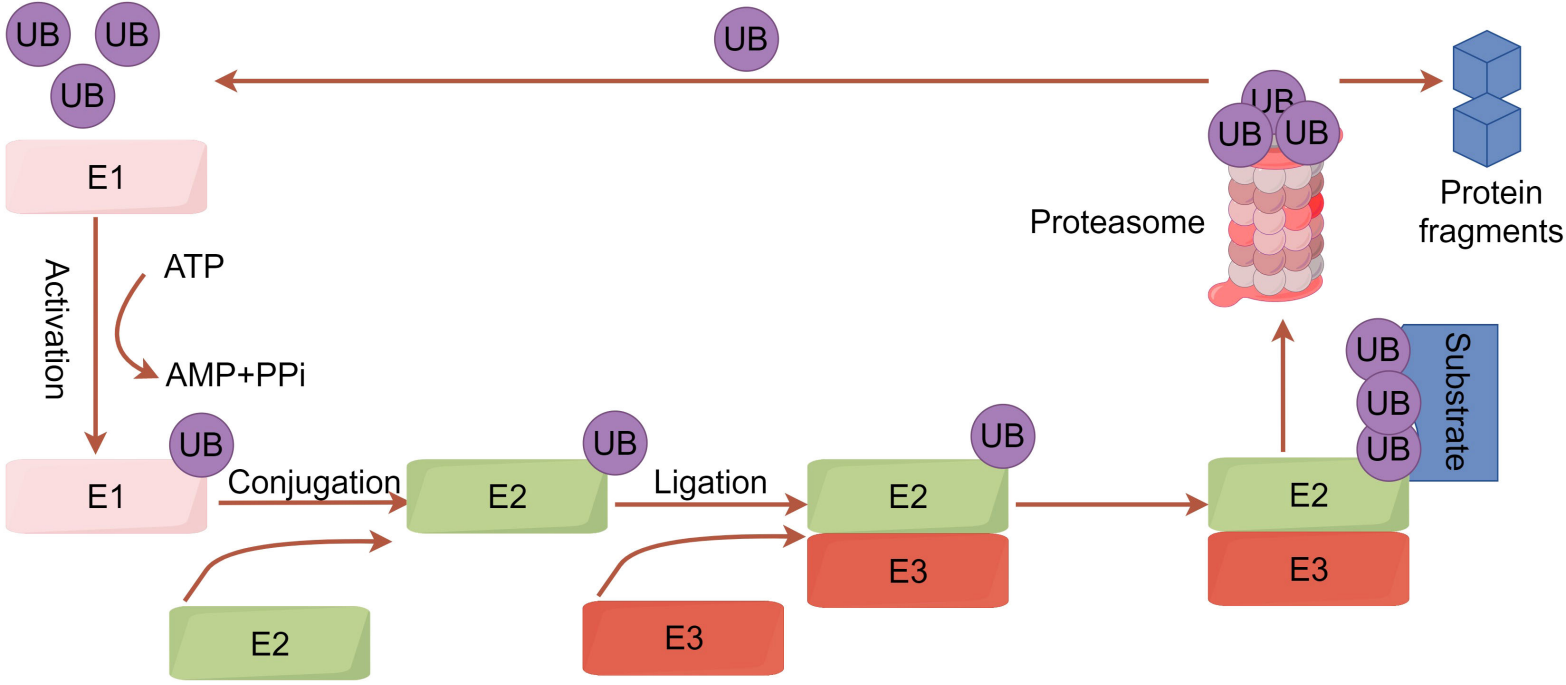
A concise overview of the ubiquitination pathway. Initially, the ubiquitin-activating enzyme E1 uses ATP hydrolysis energy to form a high-energy thioester bond between its cysteine residue (Cys) and the ubiquitin’s C-terminal glycine (Gly). Ubiquitin is then transferred to the ubiquitin-conjugating enzyme E2. Concurrently, the target protein binds to the ubiquitin ligase E3. E3 ligase links ubiquitin to specific substrates, allowing ubiquitinated proteins to be degraded into peptides by the 26S proteasome. Protein degradation occurs in the 20S proteasome, forming peptides with 3-22 amino acid residues and releasing ubiquitin molecules to re-enter the cycle.

Ubiquitination is a crucial post-translational modification of proteins in cells. Through a series of enzymatic reactions, a small protein molecule composed of 76 amino acid residues, ubiquitin, is attached to target proteins, ultimately leading to changes in the protein’s localization and function or its degradation by the proteasome (8). The ubiquitination process involves ubiquitin-activating enzyme (E1), ubiquitin-conjugating enzyme (E2), and ubiquitin ligase (E3). E1 first activates ubiquitin, which then complexes with E2. The activated ubiquitin is transferred to lysine residues on target proteins, and E3 catalyzes the covalent attachment of ubiquitin, completing the modification (9) (**Figure 1**). E3 ubiquitin ligase, the most heterogeneous enzyme in the ubiquitination process (including HECT E3s, RING E3s, and RBR E3s), plays the crucial role in recognizing target proteins and regulating the ubiquitination system (10).

Deubiquitination is the reverse process of ubiquitination, where ubiquitinated proteins release ubiquitin molecules through the action of specific hydrolytic enzymes known as deubiquitinases(DUBs) (11–13). DUBs mainly target ubiquitinated protein substrates, recognizing ubiquitin molecules or specific sequences, and dissociating ubiquitin chains. Additionally, DUBs can release ubiquitin molecules from substrates, suppress ligase activity, and thereby enhance substrate stability (2). DUBs are a diverse family of proteases, categorized into six main subfamilies based on their catalytic domains: ubiquitin-specific proteases (USPs), ubiquitin carboxyl-terminal hydrolases (UCHs), ovarian tumor proteases (OTUs), Machado-Joseph disease proteases (MJDs), motif interacting with ubiquitin-containing DUB family (MINDY), and JAMM proteases (2).

Ubiquitination and deubiquitination play crucial roles in regulating protein degradation and functionality across various cellular signaling pathways, profoundly impacting the metabolic reprogramming of cancer cells.

## 3 Lipid metabolism in cancer

Cancer cells need abundant lipids to support their rapid growth, leading to increased lipid uptake, storage, and synthesis (14). Under nutrient scarcity, cancer cells adapt their lipid metabolism to sustain survival and promote proliferation, contributing to cancer development (15). Alterations in lipid metabolism associated with cancer include increased lipid synthesis, enhanced extracellular lipid uptake in the tumor microenvironment, and improved lipid storage and mobilization within cells, particularly within lipid droplets (16). Transcription factors such as Sterol Regulatory Element-Binding Protein-1 (SREBP-1) and Peroxisome Proliferator-Activated Receptors (PPARs) promote the expression of key genes like fatty acid synthase (FASN) and stearoyl-CoA desaturase 1 (SCD1), resulting in elevated lipid synthesis, enhanced cancer cell proliferation, and unfavorable prognosis in hepatocellular carcinoma(HCC) patients (17). 3-Hydroxy-3-methylglutaryl-coenzyme A reductase (HMGCR) is a crucial enzyme in cholesterol synthesis, catalyzing the conversion of HMG-CoA to mevalonic acid. It is frequently upregulated in cancers such as gastric, brain, and prostate cancer, promoting cancer cell growth and migration. Inhibitors targeting HMGCR have been used to treat resistant solid tumors and leukemia (18). In the tumor microenvironment (TME), lipid metabolism supports both anti-tumor and pro-tumor immune responses. For instance, promoting fatty acid oxidation (FAO) can counteract the inhibitory effects of PD-1 antibodies in CD8+ T cells, while enhancing CD36 expression helps maintain the effective function and long-term survival of CD8+ T cells. However, excessive fatty acids can inhibit the anti-tumor capacity of tumor-infiltrating effector T cells (Tefs) in the TME, promoting the proliferation of regulatory T cells (Tregs) and immune suppression (19). Recent studies indicate that targeted lipid metabolism approaches have shown promising anti-cancer effects. However, relying solely on lipid metabolism reprogramming mechanisms presents several challenges in formulating cancer treatment strategies. Investigating the crosstalk between lipid metabolism and protein ubiquitination provides new insights and avenues for improving cancer treatment strategies.

## 4 Ubiquitination and lipid metabolism in cancer

Alterations in lipid metabolism are among the most significant metabolic changes in cancer cells. Dysregulation of ubiquitination of lipid metabolism proteins can also occur in the early stages of tumor formation, potentially representing a cumulative process. Ubiquitination enzymes participate in lipid metabolism and contribute to the development and progression of various types of tumors (**Table 1**).

**Table 1:** A concise overview of different Ubiquitination in various cancers.

Ubiquitin ligases	Cancer type	Lipid metabolism process	Brief biological mechanism	Refs
NEDD4	LC	Lipogenesis	ARHGEF3 reduces its acetylation on Lys17 and Lys86, leading to protein homeostasis of ACLY and dissociation between ACLY and its E3 ligase NEDD4, thereby affecting lung cancer progression	(21)
UBR4	LC	Lipogenesis	In response to high glucose levels, ACLY is acetylated at K540, K546, and K554 by lysine acetyltransferase (KAT2B), reducing the binding between ACLY and the RING-type E3 ubiquitin ligase UBR4, thereby promoting lung cancer progression.	(22)
Cullin3	LC	Lipogenesis	The E3 ligase CUL3 interacts with its adaptor protein KLHL25 to degrade ACLY in cells, thereby inhibiting lipid synthesis and growth of lung cancer cells.	(23)
COP1	HCC	Lipogenesis	The protein tyrosine phosphatase Shp2 acts as an adaptor, promoting the accumulation of phosphorylated p38 in the cytoplasm, which subsequently binds to FASN, leading to ubiquitin-mediated degradation of FASN.	(24)
TRIM21	HCC	Lipogenesis	Acetylation of FASN enhances its binding to the E3 ubiquitin ligase TRIM21. Acetylation renders FASN unstable, resulting in decreased de novo lipogenesis and tumor cell growth.	(25)
SPOP	PCa	Fatty acid synthesis	The E3 ligase SPOP regulates lipid metabolism by reducing FASN expression and fatty acid synthesis, leading to suppression of prostate cancer growth.	(26)
FBXW7β	CRC	lipogenesis	CSN6 binds to FBXW7β and FASN, antagonizing FBXW7β activity by enhancing FBXW7β self-ubiquitination and degradation. This prevents FBXW7β-mediated ubiquitination and degradation of FASN, thereby positively regulating lipogenesis and promoting colorectal cancer growth.	(27)
Hakai	CRC	Lipogenesis	Hakai regulates FASN-mediated lipid accumulation by inducing the ubiquitination and degradation of FASN via the lysosomal pathway, playing a crucial role in intestinal inflammation and cancerous bowel disease.	(28)
TRIM21	RCC	Lipogenesis	TRIM21, a E3 ligase, interacts with SREBF1 through its SPRY domain and mediates its ubiquitination and degradation via K63 linkage, suppressing lipid metabolism in renal cancer.	(30)
PHF2	HCC	Lipogenesis	ZDHHC23 mediates the palmitoylation of E3 ligase PHF2, leading to its degradation. PHF2 reduces HCC lipid generation by directly destabilizing SREBP1c.	(31)
FBXW7	HCC	Lipogenesis	DDX39B inhibits FBXW7-mediated ubiquitination and degradation of SREBP1, leading to increased lipid accumulation and promoting progression in hepatocellular carcinoma.	(32)
FBXO31	Glioma	Lipogenesis	FBXO31 downregulates SREBP1c by promoting ubiquitination and degradation of CD147, thereby inhibiting adipogenesis and tumor progression in gliomas	(33)
pVHL	RCC	Lipogenesis	pVHL ubiquitinates PPAR-γ, inhibiting ACLY expression and lipid metabolism, which is associated with renal cancer progression.	(35)
TRIM28	HCC	Lipogenesis	URI promotes the ubiquitination and degradation of p53 in a TRIM28-MDM2-dependent manner. p53 binds to the promoter of SCD1 and inhibits its transcription, thereby mediating lipid metabolism reprogramming that confers resistance to TKI-induced ferroptosis in hepatocellular carcinoma.	(40)
SKP1-Cullin-1-F-box	HCC	Lipogenesis	CAND1 regulates the reprogramming of lipid metabolism through SKP1-Cullin-1-F-box (SCF,E3 ligase)-mediated heterogeneous nuclear ribonucleoprotein A2/B1 ubiquitination, promoting hepatocellular carcinoma.	(42)
SCFβ-TRCP	HCC	Lipogenesis	E3 ubiquitin ligase SCFβ-TRCP promotes Lipin1 polyubiquitination, leading to increased protein levels and reduced triglyceride synthesis in HepG2 cells when β-TRCP is depleted.	(43)
RNF5	HCC	Cholesterol synthesis	The E3 ubiquitin ligase RNF5, an endoplasmic reticulum-anchored protein, mediates Lys29-linked polyubiquitination of SCAP, thereby activating SREBP2. RNF5 combinatorially inhibits SREBP2 activation and reduces cholesterol biosynthesis in human hepatocellular carcinoma cells.	(45)
SIAH1	LC	Cholesterol synthesis	SIAH1, an E3 ubiquitin ligase, ubiquitinates HMGCR and regulates cholesterol synthesis, thereby inhibiting lung cancer progression and enhancing drug sensitivity through cholesterol synthesis.	(46)
TRC8/RNF139	CRC	Cholesterol synthesis	The downregulation of the ubiquitin ligase RNF139 in multidrug-resistant colorectal cancer leads to the upregulation of HMGCR, thereby enhancing cholesterol synthesis.	(47)
RNF2	ESCC	Fatty acid oxidation	ETV4 transcription factor expression inhibits the expression of ubiquitin ligase RNF2, leading to increased expression of the key enzyme CPT1A involved in fatty acid oxidation, thereby playing a role in esophageal squamous cell carcinoma.	(50)
UBE2O	HCC	Fatty acid oxidation	The E2 ligase UBE2O can interact with HADHA (a mitochondrial β-oxidation enzyme) and mediate its ubiquitination and degradation, thereby regulating lipid metabolism reprogramming to promote hepatocarcinogenesis.	(51)
HUWE1	HCC	Fatty acid oxidation	Ubiquitination regulates hepatic lipid catabolism by modulating PPARα through the collaborative action of PAQR3 and HUWE1, affecting fatty acid oxidation and ketogenesis.	(52)
HRD1	TNBC	Fatty acid oxidation	HRD1 inhibits fatty acid oxidation and tumorigenesis by ubiquitinating CPT2 in triple-negative breast cancer	(54)
SIAH2	RCC/TSCC	Lipogenesis	HIF-1 activation promotes SIAH2-mediated ubiquitination and proteolysis of αKGDH. Silencing SIAH2 or mutating the ubiquitinated lysine on OGDH2 (336KA) reverses the hypoxia-induced drop in αKGDH activity, stimulates glutamine oxidation, and reduces glutamine-dependent lipid synthesis.	(55)
TRIM15	PC	Lipid droplet accumulation	The E3 ligase TRIM15 interacts with APOA1 through its PRY/SPRY domain and promotes the polyubiquitination of APOA1 via its RING domain. Enhanced degradation of APOA1 boosts lipid synthesis metabolism and facilitates lipid droplet accumulation in pancreatic cancer.	(57)

LC, lung cancer; HCC Hepatocellular carcinoma; PCA, prostate cancer; CRC colorectal cancer; RCC Renal cell carcinoma; ESCC esophageal squamous cell carcinoma; TNBC Triple-negative breast cancer; TSCC Squamous cell carcinoma of tongue; PC Pancreatic cancer ;ACLY ATP citrate lyase; FASN Fatty acid synthase; SCD1 stearoyl-CoA desaturase 1; CPT1A carnitine palmitoyltransferase 1;CPT2 carnitine palmitoyltransferase 2.

### 4.1 ACLY and cancer

The synthesis of lipids is a crucial process in lipid metabolism, providing energy substrates for the malignant proliferation of tumor cells. Adenosine triphosphate citrate lyase (ACLY) is a key enzyme linking glycolysis and lipid metabolism, catalyzing the conversion of citrate and coenzyme A into oxaloacetate and acetyl-CoA (20). In lung cancer, the deacetylation of ACLY and the ubiquitination of lysine residues are important processes for regulating protein activity and stability. ARHGEF3, a member of the Rho-GEFs family, promotes cancer cell proliferation both *in vitro* and *in vivo*. Further studies indicate that ARHGEF3 enhances the protein stability of ACLY by reducing its acetylation at Lys17 and Lys86, leading to the dissociation of ACLY from its E3 ligase NEDD4 (21). Another study found that under high glucose conditions, ACLY’s K540, K546, and K554 sites can be acetylated by histone acetyltransferase PCAF, and the acetylation levels of these three sites are enhanced in lung cancer. This acetylation reduces the binding interaction between RING-type E3 ubiquitin ligase UBR4 and ACLY, thereby maintaining ACLY-mediated protein homeostasis and leading to the enhancement of acetyl-CoA synthase and subsequent lipogenesis. Sirtuin (SIRT)2, a NAD+-dependent class III histone deacetylase, regulates the deacetylation and destabilization of ACLY, reversing the increased lipid synthesis (22). Cullin 3 interacts with ACLY through its adaptor protein Kelch-like family member 25 (KLHL25), ubiquitinating and degrading ACLY in cells. By negatively regulating ACLY, CUL3 inhibits lipid synthesis, cell proliferation, and xenograft tumor growth in lung cancer cells. Additionally, the ACLY inhibitor SB-204990 significantly mitigates the promotion of lipid synthesis induced by CUL3 downregulation (23).

### 4.2 FASN and cancer

Yu et al. found that phosphorylated COP1 accumulated in the cytoplasm of mouse livers, subsequently binding to FASN through Shp2 as an adapter. This leads to the formation of the FASN-Shp2-COP1 complex and the ubiquitin-mediated degradation of FASN (24). Additionally, Lin et al. found that FASN, through deacetylation by HDAC3, enhances its binding with the E3 ubiquitin ligase TRIM21. Deacetylation stabilizes FASN, allowing it to bind more effectively with TRIM21, thereby reducing lipogenesis and inhibiting cancer cell growth (25). The increased rate of *de novo* fatty acid synthesis in prostate cancer cells is closely related to FASN. The tumor suppressor gene speckle-type POZ protein (SPOP), which is an E3 ubiquitin ligase, regulates lipid metabolism by reducing FASN expression and fatty acid synthesis, thereby leading to tumor suppression (26).

FBXW7β is a cytoplasmic subtype of FBXW7 that is frequently mutated in CRC and serves as an E3 ligase for FASN. Wei et al. found that CSN6 binds to both FBXW7β and FASN, and antagonizes the activity of FBXW7β by enhancing its self-ubiquitination and degradation, thereby preventing FBXW7β-mediated ubiquitination and degradation of FASN, actively regulating lipogenesis. Furthermore, the EGF-regulated CSN6-FASN axis is a reason for poor prognosis in CRC. The EGF-CSN6-FASN axis promotes tumor growth, suggesting a therapeutic strategy combining Orlistat (targeting pancreatic lipase in the gastrointestinal tract, inhibiting the production of fatty acids) and Cetuximab(targeting the epidermal growth factor receptor EGFR, blocking its activation and downstream signaling pathways) (27). Another study found that the E3 ubiquitin ligase Hakai regulates FASN-mediated lipid accumulation by inducing the ubiquitination of FASN, thereby promoting its lysosome-mediated degradation, further elucidating its role in intestinal inflammation and cancerous bowel disease (28).

### 4.3 Transcriptional regulation of lipogenesis

The transcriptional regulation of lipogenesis is currently a widely studied perspective. Ubiquitinases interact with key transcription factors in lipid metabolism regulation, inhibiting or promoting the expression of key enzymes in lipid metabolism, thereby affecting the occurrence of cancer. For example, the process of adipogenesis is regulated by the SREBPs family transcription (SREBP1a and SREBP1c, SREBP2). SREBP1 mainly regulates the expression of fatty acids synthesis genes and LDLR, while SREBP2 prioritizes the expression of cholesterol biosynthesis genes (29). Renal cell carcinoma (RCC) is a metabolic disease characterized by significant alterations in the lipid profile compared to healthy tissue. TRIM21 has been identified as a novel E3 ligase for SREBP1. The SPRY domain of TRIM21 can bind to SREBP1 and mediate its ubiquitination and degradation via K63 linkage, unveiling potential avenues for targeted metabolic therapy in RCC (30). Studies (31) have found that the dietary palmitic acid levels in HCC patients may profoundly affect lipid metabolism changes. The potential mechanism involves the palmitoylation of plant homeodomain finger protein 2 (PHF2) mediated by zinc finger DHHC-type palmitoyltransferase 23 (ZDHHC23), which subsequently enhances the ubiquitin-dependent degradation of PHF2. PHF2 acts as an E3 ubiquitin ligase for SREBP1c, exerting tumor suppressor functions by directly destabilizing SREBP1c and reducing SREBP1c-dependent lipogenesis. DDX39B is an RNA helicase involved in processes such as RNA processing, transcription, and transport. Studies have found that DDX39B interacts with the E3 ubiquitin ligase FBXW7, limiting the ubiquitination of SREBP1. This interaction subsequently promotes the proliferation, migration, invasion, and lipid synthesis of HCC cells (32). FBXO31 accelerates the ubiquitination and degradation of CD147, thereby downregulating the expression of SREBP1c. Additionally, overexpression of FBXO31 leads to reduced lipogenesis by inhibiting the activation of the AKT/mTOR signaling axis, thus preventing tumor growth and invasiveness in gliomas (33).

PPARs are a family of ligand-activated nuclear transcription factors. PPARs include three subtypes: PPAR-α, PPAR-β/δ, and PPAR-γ, with PPAR-γ being the most extensively studied. It has been found that PPAR-γ plays a significant regulatory role in inflammation, atherosclerosis, insulin resistance, glucose and lipid metabolism, tumor development, and obesity (34). Von Hippel-Lindau (VHL) deficiency leads to lipid accumulation and mitochondrial dysfunction in RCC cells. Research has found that VHL directly interacts with and promotes the ubiquitination of PPARγ. Additionally, PPARγ is identified as a transcription factor that regulates ACLY expression (35).

P53 is one of the common tumor suppressor genes in human cancers. It mainly functions by regulating downstream target genes, acting as a transcription factor in the cell nucleus. p53 plays a crucial role in regulating lipid synthesis (36), fatty acid oxidation (FAO) (37), and sphingolipid metabolism (38). Thibault et al. found that p53 inhibits the expression of SCD1, converting monounsaturated phospholipids into saturated phospholipids. This conversion inhibits the oncogenic protein kinase B (AKT) pathway, thereby hindering tumor growth (39). Similarly, P53 binds to the promoter of SCD1 and inhibits its transcription, thereby mediating lipid metabolism reprogramming and developing resistance tyrosine kinase inhibitor (TKI) induced ferritic anemia in HCC. The potential regulatory mechanism of p53 is that URI promotes the ubiquitination and degradation of p53 in a TRIM28-MDM2 dependent manner (40).

Cullin-Ring E3 ubiquitin ligases (CRLs), the largest family within the UPS, play significant roles in various intracellular processes, physiology, and diseases such as cancer, with the Skp1-Cullin1-F-Box (SCF) complex being one of the most well-studied members of this family (41). Studies have found that CAND1 is associated with poor prognosis in HCC and promotes the expression of lipid synthesis genes by dissociating the SCF complex. Lipin1 is an enzyme and inhibitor in the sterol SREBP transcription factor family, capable of activating genes that encode lipogenic factors (42). In HCC cells, the SCFβ-TRCP E3 ubiquitin ligase complex targets Lipin1 for ubiquitination and degradation, leading to increased SREBP-dependent gene expression and enhanced triglyceride synthesis (43). The small molecule Z0933M can disrupt the function of the SCF E3 ligase and inhibit lung cancer growth, demonstrating that targeting the SCF complex is an effective cancer treatment approach (44).

### 4.4 Cholesterol biosynthesis

SREBP2, a key transcription factor regulating cholesterol metabolism, is activated by the SREBP chaperone SCAP. The ring finger protein 5 (RNF5), an endoplasmic reticulum-anchored E3 ubiquitin ligase, mediates the Lys29-linked polyubiquitination of SCAP, thereby activating SREBP2 to regulate cholesterol biosynthesis. Mechanistic studies indicate that RNF5 binds to the transmembrane domain of SCAP and ubiquitinates lysine 305 located in the cytosolic loop 2 of SCAP. Additionally, RNF5-mediated ubiquitination enhances the interaction between luminal loop 1 and loop 7 of SCAP, a critical event for SREBP2 activation. Through this mechanism, the ubiquitination-induced conformational change of SCAP regulates cholesterol biosynthesis (45).

HMGCR, the rate-limiting enzyme of the mevalonate pathway, is located in the endoplasmic reticulum where it catalyzes the conversion of HMG-CoA to mevalonate in cholesterol biosynthesis.Yuan et al. found that SIAH1, an E3 ubiquitin protein ligase, ubiquitinates HMGCR and influences cholesterol metabolism by regulating key enzymes in cholesterol synthesis, thereby reducing sensitivity to the drug cisplatin (46). P-glycoprotein and multidrug resistance-associated protein 1 are two membrane transport proteins involved in multidrug resistance in colorectal cancer, whose activity is increased by the high cholesterol content in the plasma membrane and detergent-resistant membranes. The potential mechanism might be the E3 ligase translocation in renal carcinoma on chromosome 8 (TRC8), also known as RNF139, which leads to a decreased ubiquitination rate of HMGCR, resulting in increased cholesterol synthesis (47).

### 4.5 Fatty acid oxidation

FAO utilizes acetyl-CoA subunits generated from fatty acids to produce nicotinamide adenine dinucleotide (NADH), acetyl-CoA, and adenosine triphosphate (ATP), supporting energy production, redox homeostasis, and biosynthetic reactions. Dysregulated FAO can enhance tumor metastasis, drug resistance, and immune evasion (48). Carnitine O-palmitoyltransferase 1 (CPT1A), a key enzyme in the FAO pathway, is located on the outer mitochondrial membrane and is responsible for transporting long-chain fatty acids from the cytoplasm into the mitochondria, which is the first step in FAO (49). CPT1A is significantly upregulated in esophageal squamous cell carcinoma (ESCC) cellines. Mechanistically, the expression of the transcription factor ETV4 inhibits the expression of the E3 ubiquitin ligase RNF2, leading to increased expression of CPT1A at both mRNA and protein levels. Additionally, genetic or pharmacological disruption of CPT1A shuts down NADPH supply, thereby preventing anchorage-independent growth of ESCC cells *in vitro* and lung metastasis in xenograft tumor models (50).

The E2 conjugating enzyme UBE2O targets HADHA (a mitochondrial β-oxidation enzyme), mediating its ubiquitination and degradation, thereby regulating lipid metabolic reprogramming and promoting the development of HCC (51). Zhao et al. found that liver-specific deletion of the PAQR3 gene reduced hepatic triglyceride levels while increasing fatty acid oxidation and ketogenesis during fasting. The underlying mechanism involves PAQR3 directly interacting with PPARα, increasing its polyubiquitination and proteasome-mediated degradation. Additionally, the E3 ubiquitin ligase HUWE1 was identified as mediating PPARα polyubiquitination (52).

Tumor development and progression are often accompanied by increased glucose and glutamine consumption as well as enhanced lipid synthesis. However, the interrelationship among glucose, glutamine, and lipid synthesis, as well as how tumor cells sense glucose and glutamine levels and regulate lipid metabolism, is not yet well understood. Recent studies have discovered that ammonia released during glutamine hydrolysis can participate in the dissociation of SCAP-Insig. Specifically, ammonia interacts with the D428, S326, and S330 amino acid residues of SCAP, altering the conformation of SCAP and leading to the dissociation of SCAP from Insig. This ultimately promotes the activation of SREBP and the lipid synthesis process, playing a significant role in tumor development and progression (53). Some studies have also found that protein ubiquitination may play a bridging role in the synthesis and breakdown of ammonia metabolism and lipid metabolism. Dependence on glutamine and accelerated FAO are metabolic characteristics of triple-negative breast cancer (TNBC). Under glutamine deprivation, which specifically inhibits the proliferation of TNBC cells, a significant downregulation of HRD1 expression was observed. HRD1 directly ubiquitinates and stabilizes CPT2 through K48-linked ubiquitination (54). Under hypoxic conditions, glutamine metabolism shifts from oxidation to reductive carboxylation. A mechanism has now been identified where HIF-1 activation leads to a significant reduction in the activity of the key mitochondrial enzyme complex α-ketoglutarate dehydrogenase (αKGDH). HIF-1 activation promotes SIAH2-mediated ubiquitination and proteolysis of the 48 kDa splice variant of the E1 subunit of the αKGDH complex (OGDH2). Knocking down SIAH2 or mutating the ubiquitination lysine residue on OGDH2 reverses the hypoxia-induced decrease in αKGDH activity, stimulates glutamine oxidation, and reduces glutamine-dependent lipid synthesis (55).

### 4.6 Lipid homeostasis

Lipid metabolic homeostasis refers to the state in which the processes of lipid synthesis, breakdown, and transport are maintained in relative balance within the body. Lipids are one of the crucial energy sources in living organisms and are also essential components of cell membrane structure and function (56). Lipid droplets are organelles composed of a phospholipid monolayer and a core of neutral lipids, providing energy for metabolic needs.TRIM15, as an E3 ligase, has been found to be associated with lipid homeostasis. It interacts with APOA1 through its PRY/SPY domain and promotes polyubiquitination of APOA1 through its RING domain. The degradation of APOA1 enhances the lipid anabolism of pancreatic cancer cells and promotes the accumulation of lipid droplets (57).

## 5 DUBs and lipid metabolism in cancer

### 5.1 ACLY/FASN and cancer

Deubiquitinating enzymes, like ubiquitinating enzymes, often interact with key metabolic enzymes when regulating fatty acid synthesis. DUBs act as regulators of lipid metabolism and play essential roles in various types of tumors (**Table 2**). The ubiquitin-specific peptidase 2a(USP2a) interacts with and stabilizes FASN, which is typically overexpressed in biologically aggressive human tumors. Additionally, USP2a is androgen-regulated and overexpressed in prostate cancer, and its functional inactivation leads to a reduction in FASN protein and an increase in apoptosis (58). The deubiquitinase USP30 is abundant in HCC that occurs in mice maintained on a high-fat diet. IKKβ phosphorylates and stabilizes USP30, promoting USP30 to deubiquitinate ATP citrate lyase ACLY and FASN. IKKβ also directly phosphorylates ACLY, promoting the interaction between USP30 and ACLY and the deubiquitination of the latter (59).

**Table 2:** A concise overview of different DUBs in various cancers.

DUBs	Cancer type	Lipid metabolism process	Brief biological mechanism	Refs
USP2a	PCa	Fatty acid synthesis	USP2a interacts with FASN and stabilizes it, promoting the progression of prostate cancer.	(58)
USP30	HCC	Fatty acid synthesis	IKKβ phosphorylates and stabilizes USP30, promoting USP30-mediated deubiquitination of ACLY and FASN induction, leading to the occurrence of hepatocellular carcinoma.	(59)
USP22	CRC	Fatty acid synthesis	The ROS-mediated inhibition of USP22 is relieved, leading to the stabilization of FASN, thereby promoting lipid synthesis and colorectal cancer growth.	(61)
USP19	CRC	Lipogenesis	USP19 antagonizes RNF1-mediated degradation of ME1 through deubiquitination, thereby promoting lipid metabolism and NADPH production while suppressing ROS, consequently triggering the progression of colorectal cancer.	(62)
USP7	HCC	de novo lipogenesis	The USP7/ZNF638 axis selectively increases the cleavage of SREBP1C through AKT/mTORC1/S6K signaling, forming a USP7/ZNF638/SREBP1C nuclear complex. This complex regulates lipogenic enzymes, contributing to the progression of lipogenesis-related hepatocellular carcinoma	(63)
USP7	CCA	Fatty acid synthesis	Under hypoxic conditions, SKA3 recruits PARP1 to bind with HIF-1α, thereby enhancing USP7-mediated deubiquitination of HIF-1α, promoting the proliferation and fatty acid synthesis of cholangiocarcinoma cells.	(64)
USP7	HNSC	de novo lipogenesis	USP7 and PRMT5-dependent G3BP2 stability drives de novo lipogenesis and tumorigenesis in head and neck squamous cell carcinoma.	(65)
UCHL1	BC	Fatty acid synthesis	UCHL1 may promote Doxorubicin resistance in breast cancer by upregulating the synthesis of free fatty acids.	(67)
USP22	HCC	Fatty acid synthesis	USP22 deubiquitinates and enhances the stability of PPARγ protein, thereby promoting the synthesis of FASN, ACLY, and ACACA, and facilitating the malignant progression of hepatocellular carcinoma.	(69)
USP1	PCa	Fatty acid synthesis	USP1 enhances the stability of CCAAT/enhancer-binding protein β (C/EBPβ) and accelerates lipid formation and lipid accumulation in prostate cancer.	(71)
SLP2	HCC	Lipogenesis	SLP2 affects the ubiquitin-proteasome degradation pathway of JNK2, thereby maintaining the protein stability of JNK2 and increasing the activity of SREBP1, which impacts the progression of hepatocellular carcinoma and lipid synthesis.	(73)
USP28	LC	Mevalonate metabolism	USP28 deubiquitinates SREBP2 to regulate mevalonate metabolism and promote the progression of lung squamous cell carcinoma.	(75)
USP35	PCa	Mevalonate metabolism	USP35 can directly deubiquitinate and stabilize the BRPF1 protein. Elevated BRPF1 can bind to the promoter of SREBP2 and activate the transcriptional capacity of SREBP2, thereby promoting the expression of metabolic characteristics associated with mevalonate metabolism.	(76)
USP14	GC	Fatty acid oxidation	The activation of USP14 determines the increased stability of the SIRT1 protein and is essential for the activation of fatty acid oxidation and the immunosuppressive phenotype in macrophages derived from gastric cancer.	(78)
USP18	LC	Fatty acid oxidation	Increased expression of USP18, by reducing ubiquitination, enhances UCP1 protein levels, thereby promoting fatty acid β-oxidation and driving tumorigenesis.	(79)

PCa, Prostate cancer; HCC Hepatocellular carcinoma; CRC, Colorectal cancer; CCA Cholangiocarcinoma; HNSC Head and neck squamous cell carcinoma; BC Breast cancer; LC Lung cancer; GC Gastric cancer ; ACLY ATP citrate lyase; FASN Fatty acid synthase; NADPH Nicotinamide Adenine Dinucleotide Phosphate.

Oxidative stress is a physiological state in which there is an imbalance between oxidative and antioxidant effects in the body. Unsaturated fatty acids in oxidized lipids, as the material basis, are oxidized under oxidative stress to form oxidative metabolites, thereby affecting life activities (60).

Excessive production of reactive oxygen species (ROS) and abnormal lipid metabolism are established hallmarks of cancer. However, the role of ROS in lipid synthesis during tumorigenesis is almost unknown. Studies have found that ROS regulates lipid synthesis and thereby controls colorectal tumorigenesis through a p53-dependent mechanism. In p53 wild-type CRC cells, hydrogen peroxide (H2O2)-induced p53 expression suppresses the transcription of the USP22, which otherwise deubiquitinates and stabilizes FASN, thereby inhibiting fatty acid synthesis. In p53-deficient CRC cells, ROS-mediated inhibition of USP22 is alleviated, leading to the stabilization of FASN, thereby promoting lipid synthesis and tumor growth (61). Zhu et al. found that ERK2 phosphorylates ME1 at T103, thereby inhibiting its polyubiquitination and proteasomal degradation, and enhancing its interaction with USP19. USP19 counteracts RNF1-mediated degradation of ME1 through deubiquitination, thereby promoting lipid metabolism and NADPH production while inhibiting ROS. Simultaneously, ROS significantly increases PD-L1 mRNA levels by accelerating the expression of the transcription factor NRF2.ERK2 inhibitors in combination with anti-PD-L1 antibodies significantly inhibit CRC development (62).

Recent studies suggest that USP7 plays a role in lipid synthesis in cholangiocarcinoma(CCA) and head and neck squamous cell carcinoma(HNSC).USP7 has been shown to interact with and deubiquitinate ZNF638, and it can also promote the transcription of ZNF638 by stabilizing cAMP response element-binding protein (CREB). The USP7/ZNF638 axis selectively increases the cleavage of SREBP1C through AKT/mTORC1/S6K signaling, forming a USP7/ZNF638/SREBP1C nuclear complex. This complex regulates lipogenic enzymes, including acetyl-CoA carboxylase (ACACA), FASN, and SCD. In a fructose-induced mouse model of hepatic steatosis, the elimination of USP7 or ZNF638 significantly improved disease progression. Additionally, the USP7/ZNF638 axis is involved in the progression of lipogenesis-related HCC (63). Under hypoxic conditions, SKA3 recruits PARP1 to bind with HIF-1α, thereby enhancing the poly-ADP-ribosylation (PARylation) of HIF-1α. This PARylation enhances the interaction between HIF-1α and USP7, triggering the deubiquitination of HIF-1α under hypoxic conditions. Additionally, both PARP1 and HIF-1α are upregulated in CCA and promote the proliferation of CCA cells (64). As early as 2016, Liu et al. discovered that protein arginine methyltransferase 5 (PRMT5) is a binding partner of SREBP1a and symmetrically dimethylates it at R321, thereby promoting its transcriptional activity. Additionally, PRMT5-induced methylation prevents GSK3β from phosphorylating SREBP1a at S430, leading to its dissociation from FBXW7 and escaping degradation through the ubiquitin-proteasome pathway. Consequently, this methylation stabilizes SREBP1a, increasing *de novo* lipogenesis both *in vivo* and *in vitro*, and accelerates cancer cell growth (17). PRMT5-mediated G3BP2-R468me2 enhances its binding with the deubiquitinase USP7, thereby ensuring the deubiquitination and stabilization of G3BP2 and the activation of ACLY, which stimulates *de novo* lipogenesis and tumorigenesis (65).

Ubiquitin carboxyl-terminal hydrolase L1 (UCHL1) is expressed in neuronal cytoplasm, testes, and ovaries. It is a protein released into plasma during neuronal damage and has been extensively studied in various neurodegenerative diseases such as Parkinson’s disease and Alzheimer’s disease (66). UCHL1 may promote doxorubicin (DOX) resistance in breast cancer (BC) by upregulating free fatty acid synthesis, as evidenced by reduced expression of FFA synthase and restored DOX sensitivity following UCHL1 inhibition (67). Another study also found that UCHL1 inhibits lipid accumulation and foam cell formation by promoting the degradation of CD36 protein, indicating that UCHL1 may be a potential target for atherosclerosis treatment (68).

### 5.2 Transcriptional regulation of lipogenesis

PPARγ is a key regulator of lipid metabolism, particularly in promoting the uptake, storage, and metabolism of fatty acids. It influences lipid synthesis and breakdown by regulating the expression of lipid metabolism-related genes. In this study, USP22 was identified as a critical regulator of fatty acid synthesis. USP22 directly interacts with and deubiquitinates PPARγ, which is stabilized by K48-linked deubiquitination. This stabilization further enhances the expression of ACACA and ACLY. Additionally, it was found that USP22 promotes *de novo* synthesis of fatty acids and contributes to the development of HCC (69). The regulation of lipid synthesis by USP22 involves not only transcriptional levels but also direct deubiquitination of key enzymes, demonstrating that USP22 is an important regulator of lipid metabolism and a potential therapeutic target.

The CCAAT/enhancer-binding protein family (C/EBP-α, -β, and -δ) and PPARs play key roles in adipocyte differentiation and adipogenesis. During the early stages of adipocyte differentiation, C/EBP-β and C/EBP-δ are induced, which subsequently transactivate the expression of PPARγ and C/EBP-α (70). The expression of USP1 is significantly upregulated during adipocyte differentiation and in the adipose tissue of high-fat diet (HFD) mice. USP1 directly deubiquitinates C/EBPβ and increases its protein expression, leading to adipogenesis and lipid accumulation. Oral administration of ML323(USP1 inhibitor) to HFD-fed mice resulted in weight loss and improved insulin and glucose sensitivity. Following ML323 treatment, both the amount of fat and the size of adipocytes in white adipose tissue were significantly reduced, along with a decrease in the expression of genes involved in adipogenesis and inflammatory responses (71). Lee et al. found that among various mouse tissue mRNA samples, USP1 expression levels are higher in adipose tissue than in other tissues. When USP1 is knocked down, all adipogenic transcription factors (PPARγ and C/EBP-α and β, FASN, and fatty acid-binding protein 4) are downregulated. USP1 may play an important role in the process of adipogenesis (72).

SLP2 is a protein localized in the mitochondria and has been shown to be involved in mitochondrial biogenesis. Studies have demonstrated that SLP2 can bind to the C-terminus of JNK2. This interaction affects the ubiquitin-proteasome degradation pathway of JNK2, thereby indirectly regulating its degradation. As a result, SLP2 helps maintain the stability of JNK2 protein, which in turn enhances the activity of SREBP1 (73).

### 5.3 Cholesterol biosynthesis

Cholesterol is an essential lipid whose synthesis is both nutritionally and energetically expensive. In mammals, cholesterol biosynthesis increases after feeding and is suppressed under fasting conditions. However, the regulatory mechanisms governing cholesterol biosynthesis during the fasting-feeding transition remain largely unknown. USP20 stabilizes HMGCR, the rate-limiting enzyme in the cholesterol biosynthesis pathway, in the fed state. Postprandial increases in insulin and glucose levels stimulate mTORC1 to phosphorylate USP20 at S132 and S134; USP20 is recruited to the HMGCR complex and counteracts its degradation. In liver-specific USP20 knockout mice and USP20(S132A/S134A) knock-in mice, the feeding-induced stability of HMGCR is abolished (74).

SREBP2 is a major regulator of the mevalonate pathway (MVP), a biosynthetic process that drives the synthesis of polyterpenoids, heme A, ubiquinone, and cholesterol, and provides substrates for protein isoprenylation. Studies have identified SREBP2 as a novel substrate of USP28, a deubiquitinase frequently upregulated in lung cancer. Silencing USP28 reduces the expression of MVP enzymes and decreases the metabolic flux into this pathway. It has also been demonstrated that USP28 binds to mature SREBP2, leading to its deubiquitination and stabilization. Depletion of USP28 makes cancer cells highly sensitive to statin inhibition of MVP, and this inhibition can be rescued by the addition of geranylgeranyl pyrophosphate. Statins synergize with dual USP28/25 inhibitors to reduce the viability of SCC cells (75). USP35 directly deubiquitinates and stabilizes BRPF1 protein. Accumulated BRPF1 accelerates cell growth, stem cell-like properties, and *in vitro* and *in vivo* migration. Interestingly, high levels of BRPF1 can bind to the promoter of SREBP2 and activate its transcriptional capability. Thus, the USP35/BRPF1 axis promotes the expression of mevalonate (MVA) metabolic characteristics in an SREBP2-dependent manner. USP35 relies on BRPF1 to maintain the activity of mevalonate metabolism in PRAD cells. Finally, targeting BRPF1 or using MVA inhibitors (such as atorvastatin) effectively inhibits *in vivo* tumor growth in USP35-high PRAD models (76).

### 5.4 Fatty acid oxidation

Stromal cells and immune cells within the TME also undergo lipid metabolism reprogramming, impacting tumor functional phenotypes and immune responses. Given the significant role of lipid metabolism in supporting tumor progression and reshaping the tumor microenvironment, targeting lipid metabolic pathways may offer new avenues for cancer therapy (77). USP14 plays a broad role in tumor malignancy and fat metabolism regulation. However, there are gaps in researchers’ understanding of its substrates, making deubiquitinase a challenging clinical target. Under the control of IU1 and FAO inhibitors, tumor-associated macrophages (TAMs), particularly M2 macrophages, were isolated from tumor cell lines or polarized from primary THP1 cells. Cytokine-controlled macrophages were compared to assess their ability to induce USP14 expression. Macrophage metabolism was analyzed using fatty acid uptake assays and oxygen consumption rate (OCR) measurements. USP14 was found to be associated with poor tumor prognosis and an unfavorable immune phenotype in gastric cancer patients and mouse tumor models. The activation of USP14 determined the increased stability of SIRT1 protein and was necessary for macrophage fatty acid oxidation and the activation of an immunosuppressive phenotype. Although the overexpression of USP14 alone was insufficient to polarize macrophages to an M2 phenotype, inhibiting USP14 with IU1 in tumor-bearing mice disrupted the suppressive activity of pro-tumor macrophages and effectively remodeled the immune microenvironment characteristics (78). USP18-deficient mice (compared to wild-type mice) exhibit lower lipolysis rates, altered fat-to-body weight ratios, and cold sensitivity. Previous studies have shown that USP18 promotes lung tumorigenesis. The study aimed to determine whether USP18 affects lipid and fatty acid metabolism. It was found that the deletion of USP18 inhibited the expression of adipose triglyceride lipase (ATGL), while increased expression of USP18 upregulated ATGL in cancer cells. An E1-like ubiquitin-activating enzyme promoted ISG15 conjugation and destabilization of ATGL. Immunoprecipitation analysis confirmed covalent binding of ISG15 to ATGL. The protein expression of thermogenic regulators was detected in the brown adipose tissue of USP18-null and wild-type mice. Uncoupling protein 1 (UCP1) was suppressed in USP18-deficient fat. Increased USP18 expression enhanced UCP1 protein expression in lung cancer cell lines by reducing ubiquitination, and the increase in UCP1 expression enhanced cell proliferation. UCP1 knockdown inhibited proliferation. β-Hydroxybutyrate colorimetric assays conducted after acquiring UCP1 expression showed increased cellular fatty acid β-oxidation, which also increased fatty acid β-oxidation in Seahorse assays. These findings suggest that USP18 is a pharmacological target for controlling fatty acid metabolism (79).

## 6 Future directions

This review briefly discusses the role of ubiquitination and deubiquitination in cancer lipid metabolism, highlighting how E3 ligases and DUBs participate in lipid synthesis, cholesterol synthesis, transcriptional reprogramming, and lipid homeostasis by regulating metabolic enzymes, transcription factors, and related signaling pathways. Most studies focus on the direct involvement of ubiquitination and deubiquitination in metabolic enzymes, which provides potential for jointly developing ubiquitin inhibitors and metabolic enzyme inhibitors. However, the regulatory factors of ubiquitin ligases and deubiquitinases remain unclear, and the E3 ligase/DUB-substrate network is highly complex. A single E3 ligase or DUB can target multiple substrates, meaning their roles are context-dependent. Their specific roles in cancer may vary depending on substrates, tissue types, tumor stages, or different metabolic conditions. Additionally, one molecule can be regulated by multiple E3 ligases or DUBs. In conclusion, ubiquitination and deubiquitination are considered crucial regulators of lipid metabolic reprogramming in cancer cells, warranting further research to improve cancer treatment.
